# The multidimensional regulation roles and mechanisms of calcium in fruit quality

**DOI:** 10.1111/jipb.70192

**Published:** 2026-02-16

**Authors:** Fei Jiang, Siyang Gao, Mengdi Li, Zeqi Zhao, Chengwei Yang, Ji‐Hong Liu, Chunlong Li

**Affiliations:** ^1^ National Key Laboratory for Germplasm Innovation & Utilization of Horticultural Crops College of Horticulture and Forestry Sciences Huazhong Agricultural University Wuhan 430070 China; ^2^ Hubei Hongshan Laboratory Wuhan 430070 China

**Keywords:** calcium, fruit quality, regulation mechanisms, signaling

## Abstract

Calcium (Ca^2+^), a dual‐functional mineral that serves both as an essential structural factor and a signaling molecule, plays a critical role in regulating fundamental physiological processes in plants, including development, stress response, and fruit quality traits. However, a comprehensive and systematic summary of calcium's regulatory functions in fruit quality is still lacking. This review aims to clarify the pivotal roles of calcium in regulating key fruit quality attributes, including external traits such as morphology and coloration; internal nutritional properties, such as flavor‐related metabolites and bioactive compounds; and physiological disorders such as cracking, softening, browning, chilling injury, blossom‐end rot, water core, and bitter pit. Considering its diverse regulatory functions, genetic manipulation of Ca^2+^ signaling pathways and the application of nano‐calcium formulations offer promising strategies for improving fruit yield and quality in commercial production systems. This review further outlines the underlying mechanisms through which calcium influences fruit quality and suggests future research directions to address existing knowledge gaps.

## Graphical abstract

This review explores how calcium signaling molecule integrates plant hormones, environmental cues, and developmental signals to influence external fruit traits, internal nutritional properties, and physiological disorders.

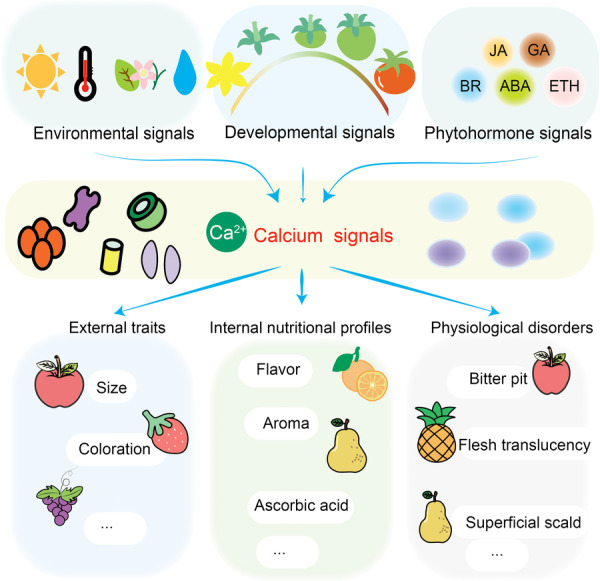

## INTRODUCTION

Global population expansion is accelerating toward 10 billion by 2050, intensifying the challenge of ensuring adequate access to nutritious food and maintaining dietary balance worldwide (Food and Agriculture Organization of the United Nations, 2017). While previous research has predominantly focused on cereal crops as staple carbohydrate sources, horticultural crops, such as vegetables, fruit trees, and ornamental plants, and medicinal species have been comparatively overlooked. Enhancing research on horticultural crops is essential, particularly nutrient‐rich fruits, which are economically important plant organs that significantly impact global human health (Wallace et al., 2019; Del Río‐Celestino and Font, 2020).

**Figure 1:**
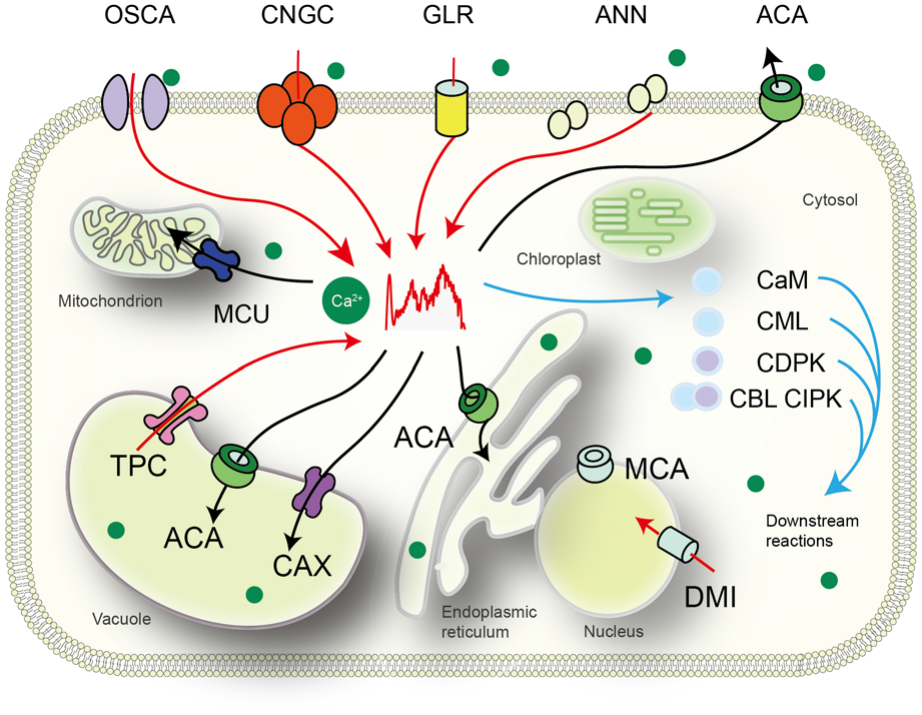
Plants employ calcium channels, pumps, exchangers, and sensors to regulate calcium signal dynamics Influx of calcium (Ca^2+^) ions is mediated by diverse channel families, including cyclic nucleotide‐gated channels (CNGCs), glutamate receptor–like channels (GLRs), hyperosmolarity‐gated channels (OSCAs), two‐pore channels (TPCs), and annexins (ANNs). Counterbalancing these influx pathways, Ca^2+^ extrusion is achieved through autoinhibited Ca^2+^ ATPases (ACAs) coupled with proton gradients, Ca^2+^/proton (H^+^) exchangers (CAXs), and specialized transporters such as mechanosensitive Ca^2+^ channels (MCAs) and symbiosis‐related Does Not Make Infections (DMI) systems. This coordinated network maintains resting cytosolic Ca^2+^ concentrations around 100 nM while enabling dynamic Ca^2+^ signaling through precisely controlled spatiotemporal patterns, including amplitude variation, frequency modulation, and distinct spatial propagation characteristics across different cellular and tissue contexts. Decoding the Ca^2+^ signal relies on an evolutionarily conserved sensor network. Highly conserved Ca^2+^‐binding proteins calmodulins (CaMs) and plant‐specific CaM‐like proteins (CMLs) directly bind Ca^2+^ ions. Ca^2+^‐dependent protein kinases (CDPKs or CPKs) perceive fluctuations in Ca^2+^ levels via their intrinsic EF‐hand domains, while calcineurin B‐like proteins (CBLs) complexed with CBL‐interacting protein kinases (CIPKs) translate Ca^2+^ signals into phosphorylation cascades through allosteric regulation.

The growth and development of horticultural crops are regulated by multiple nutritional elements, among which calcium ions (Ca^2+^) exhibit dual functionality as both an essential structural factor (Hocking et al., 2016) and a signaling molecule (Kudla et al., 2018). Indeed, calcium is involved in a large number of biological processes, including root hair development (Zhu et al., 2025), pollen tube guidance (Pei et al., 2024), stomatal guard cell movement (Shimazaki et al., 2007), circadian rhythm (Shimazaki et al., 2007), stress tolerance (Feng et al., 2023; Zhang et al., 2024b), fruit development and ripening (Gao et al., 2019), and the determination fruit quality traits (Hocking et al., 2016). Structurally, Ca^2+^ stabilizes cell walls through pectin crosslinking and membrane stabilization, directly impacting fruit firmness and postharvest quality (Atmodjo et al., 2013; Ciccarese et al., 2013; Zhang et al., 2019b). As a universal signaling mediator, calcium signaling starts with diverse stimuli, including abiotic stresses such as drought and salinity, biotic stresses such as pathogen infections, and developmental signals such as plant hormones (Tian et al., 2020). These stimuli then convert into oscillatory cytosolic Ca^2+^ signals through membrane‐localized channels, pumps, and exchangers, including hyperosmolality‐gated calcium‐permeable channels (OSCAs), glutamate receptor‐like proteins (GLRs), and cyclic nucleotide‐gated channels (CNGCs), two‐pore channels (TPCs), annexins (ANNs), calcium/proton exchangers (CAXs), and autoinhibited calcium ATPases (ACAs) (Chen et al., 2016, Tian et al., 2020, Xu et al., 2022). These spatiotemporal calcium signals are decoded by calcium‐sensing proteins, including calmodulins (CaMs), calmodulin‐like proteins (CMLs), calcineurin B‐like proteins (CBLs), and their interacting protein kinases (CIPKs), as well as calcium‐dependent protein kinases (CDPKs), thereby regulating gene expression and downstream effector activities that control essential physiological processes (Luan and Wang, 2021) (Figure 1). These calcium sensors regulate fruit quality by modulating two primary classes of target proteins. The first class includes transcription factors, such as MYB (Yu et al., 2020b), ERF (Zhu et al., 2024b), and ABI5 (Mao et al., 2025), which orchestrate gene expression networks. The second class comprises functional proteins, including SUN (Clevenger et al., 2015), UDP‐glucosyltransferase (UGT1) (Peng et al., 2016), Tonoplast Sugar Transporters (TSTs) (Li et al., 2024c), Fatty Acid Desaturase 2 (FAD2) (Liu et al., 2024), GDP‐L‐galactose phosphorylase 2 (GGP2) (Yin et al., 2024), and 4‐coumarate‐CoA ligase 2 (4CL2) (Li et al., 2022a), which directly influence fruit development and metabolic pathways. Together, these two types of targets act synergistically to control fruit quality formation.

During fruit development and maturation, calcium primarily orchestrates three key aspects, encompassing external traits such as morphological characteristics and color patterning (Zhai et al., 2019; Zheng et al., 2022), internal nutritional profiles exemplified by flavor metabolites and functional compounds (Di et al.,2024; Mao et al., 2025), and physiological disorders mechanisms addressing cracking, softening, browning, chilling injury, watercore, bitter pit, blossom‐end rot, and spongy tissue (Han et al., 2022; Mishra et al., 2022; Yang et al., 2023; Hou et al., 2023; Shu et al., 2025). Although existing research has extensively elucidated the roles of calcium in model plants, its systematic influence on key quality parameters during fruit development and maturation in horticultural crops remains insufficiently understood. This review systematically examines calcium‐mediated molecular mechanisms underlying fruit quality formation, providing theoretical insights for advances in molecular breeding and optimized calcium management strategies.

## CALCIUM‐MEDIATED REGULATION OF EXTERNAL TRAITS OF HORTICULTURAL FRUIT

### Calcium and fruit morphological characteristics

**Figure 2:**
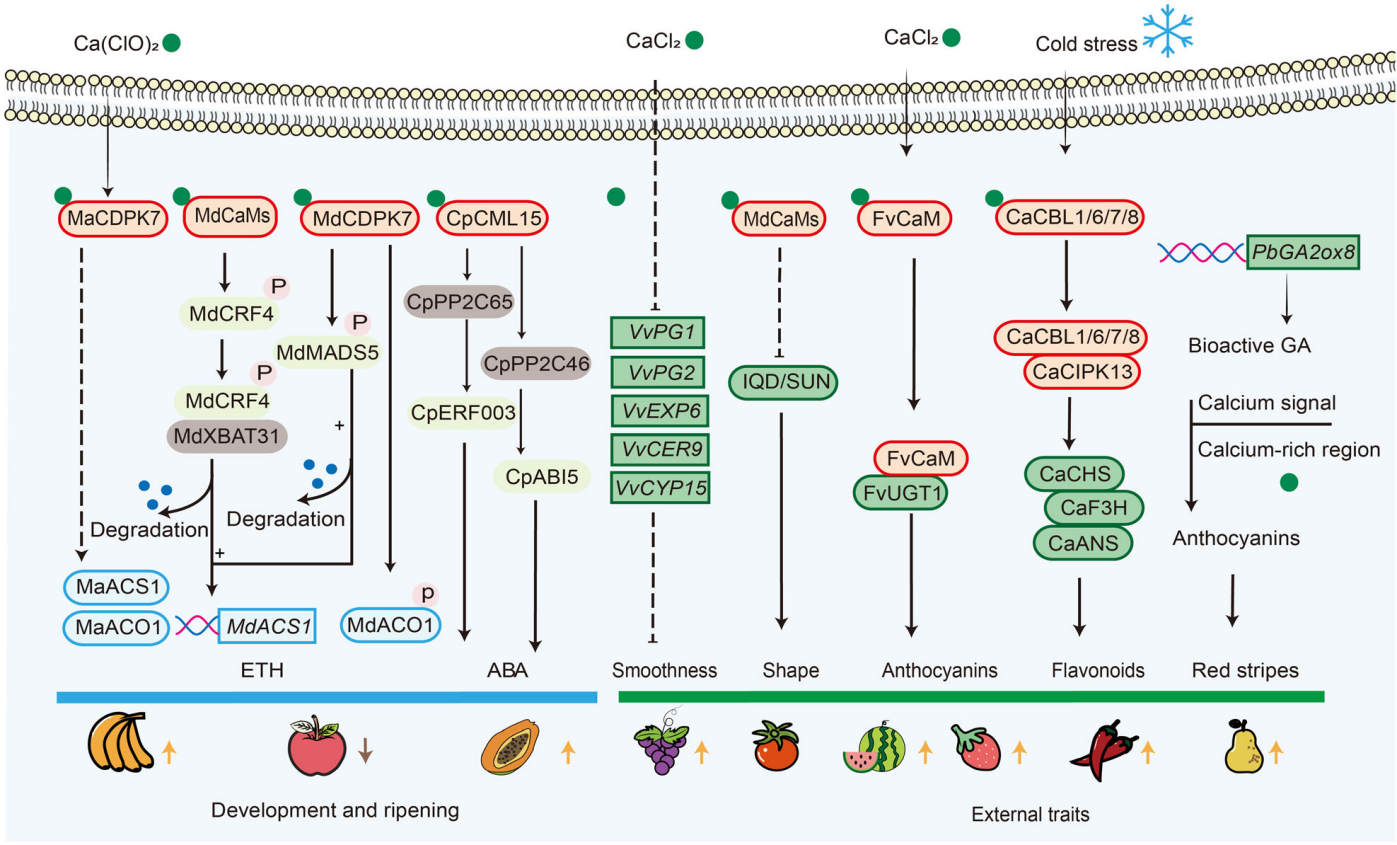
Calcium ions regulate fruit development and ripening, and also modulate external traits Ca^2+^ ions modulate fruit development and ripening through the regulation of abscisic acid (ABA) and ethylene biosynthesis. Additionally, Ca^2+^‐mediated pathways determine fruit shape, while concurrently influencing anthocyanin biosynthesis and/or compartmentalization, and fruit smoothness. ACO1, ACC oxidase 1; ACS1, 1‐aminocyclopropane‐1‐carboxylic acid (ACC) synthase 1; ANS, Anthocyanidin synthase; CER9, ECERIFERUM (E3 ubiquitin ligase); CHS, Chalcone synthase; CYP15, Cytochrome P450 member 15 (cuticular lipid biosynthesis); EXP6, Expansin 6; F3H, Flavanone 3‐hydroxylase; IQDs, IQ67‐domain proteins; MdXBAT31, MdXB3 ORTHOLOG 1 in *Arabidopsis thaliana* (E3 ubiquitin ligase); OFPs, Ovate Family Proteins; PGP, Polygalacturonase; PP2Cs, Type 2C protein phosphatases; TRMs, TONNEAU 1 Recruitment Motifs; UGT1, dUDP‐glycosyltransferase 1. Key symbols: Ub, ubiquitination; P, phosphorylation; upward arrow (↑), promotion or activation; downward arrow (↓), inhibition or suppression. Fruit drawings were drawn by the author using Adobe Illustrator, based on reference images available online.

Fruit development and ripening play a critical role in determining yield and quality. The most prominent transformations during these processes are the changes in morphological features and color patterns. The formation of fruit morphological characteristics depends on the coordinated regulation of cell division and expansion during the early stages of development, with the underlying mechanism involving the integration of complex, multidimensional signaling networks within cells (Sablowski, 2016; Janocha and Lohmann, 2018; Zhang et al., 2025b). These networks precisely regulate critical developmental processes, including cell division rate, expansion magnitude, and differentiation direction, ultimately determining fruit size, shape, and weight. The calcium signal may play a crucial role in these regulatory mechanisms (Figure 2; Table 1). Studies have demonstrated that CDPKs regulate fruit ripening in tomato (*Solanum lycopersicum*) (Leclercq et al., 2004), strawberry (*Fragaria × ananassa*) (Llop‐Tous et al., 2002), and banana (*Musa acuminata*) (Wang et al., 2017; Li et al., 2019) through the phosphorylation of target proteins. For example, MaCDPK7 in banana regulates ethylene production by directly phosphorylating the 1‐aminocyclopropane‐1‐carboxylic acid synthase (ACS) or indirectly by phosphorylating transcription factors/signaling proteins to induce ethylene synthesis gene expression. CaMs may influence ripening in tomato (Arhondakis et al., 2016), banana (Du et al., 2016; Jiang et al., 2018), papaya (*Carica papaya*) (Angel Huerta‐Ocampo et al., 2012), litchi (*Litchi chinensis*) (Jiang et al., 2017), and peach (*Prunus persica*) (Jiang et al., 2020) by activating downstream target proteins. In particular, the Ca^2+^/CaM complex directly binds to and activates cell wall‐degrading enzymes, resulting in fruit softening in peach (Jiang et al., 2020; Li et al., 2022b). Additionally, the CBLs‐CIPKs complex plays a crucial role in fruit ripening in grape (*Vitis vinifera*) (Cuellar et al., 2013) and cherry tomato (*S. lycopersicum* var. *cerasiforme*) (Tang et al., 2020) via phosphorylation cascades. Specifically, the study indicates that the widespread upregulation of *CBL* and *CIPK* genes during postharvest storage drives the ripening and senescence process of cherry tomato fruit by coordinately regulating energy metabolism and secondary metabolic networks (Tang et al., 2020).

**Table 1:** Key factors involved in calcium‐mediated regulation of horticultural fruit development and external traits

Species	Components	Affected trait	Characteristic	Reference
*Musa AAA Group “Cavendish”*	CDPK7	Development and ripening	ETH induces the expression of *CDPK7*, which in turn induces the expression of *ACO1* and *ACS1*, thereby promoting fruit ripening.	(Wang et al., 2017)
*Malus domestica*	CDPK7	Development and ripening	Calcium activates the CDPK7‐MADS5 protein degradation pathway, significantly inhibiting ethylene biosynthesis and apple fruit ripening.	(Xu et al., 2023)
*M. domestica*	CaMs‐CRF4	Development and ripening	Calcium activates the CaM‐mediated degradation pathway of the CRF4 to inhibit ethylene biosynthesis and apple fruit ripening.	(Li et al., 2024b)
*Carica papaya*	CML15‐PP2Cs	Development and ripening	Calcium activates the CML15‐mediated signal integration pathway, effectively accelerating fruit ripening.	(Zhu et al., 2024b)
*Fragaria ananassa*	FaAnn5a /5b/8	Development and ripening	Spraying whole fruit with CaCl_2_ at the postharvest white stage differentially regulates *ANN* expression and partially suppresses ripening progression.	(Chen et al., 2016)
*Vitis vinifera*	PG1/2/EXP6/CER9	Smoothness	Spraying whole grapevines with CaCl_2_ during the preharvest fruiting season activates the calcium‐induced cell wall remodeling pathway, effectively improving berry skin smoothness.	(Martins et al., 2020)
*Cucumis sativus*	IQDs‐OFPs‐TRMs	Morphology	Plant OFPs interact with TRM and IQD proteins to regulate cytoskeleton activity and cell division, integrating hormonal and stress signals to shape organs.	(Snouffer et al., 2020)
*Citrullus lanatus*	FS1	Morphology	The watermelon shape gene *Cla011257* encodes an IQD protein; its 159 bp deletion truncates the protein, co‐segregates with elongated fruit, and likely regulates cell arrangement via calcium signaling.	(Dou et al., 2018)
*V. vinifera*	SUN	Morphology	*SUN* regulates auxin signaling and cell division, affecting longitudinal cell number and arrangement to promote fruit elongation.	(Zheng et al., 2022)
*Solanum lycopersicum*	SUN	Morphology	The tomato *SUN* gene, encoding a calmodulin‐binding IQ protein, influences cell division and patterning pathways to alter axial cell number and elongate fruit shape.	(Clevenger et al., 2015)
*F. ananassa*	UGT1	Anthocyanin	Foliar spraying of CaCl_2_ before fruit ripening promotes CaMs binding to UGT1, relieves substrate inhibition, enhances glycosylation of anthocyanin precursors, and accelerates anthocyanin accumulation.	(Peng et al., 2016)
*Capsicum annuum*	CBL1/6/7/8‐ CIPK13	Anthocyanin	CIPK13 interacts with CBL1/6/7/8, enhancing ROS scavenging enzyme activity and promoting anthocyanin accumulation in pepper and tomato.	(Ma et al., 2021)
*Pyrus bretschneideri*	GA2OX8	Anthocyanin	Calcium signaling regulates anthocyanin genes via the GA2ox8‐DELLA pathway, causing anthocyanins to accumulate mainly in calcium‐rich areas near veins and form red stripes.	(Zhai et al., 2019)

Calcium regulates developmental and ripening processes, with its endogenous levels and exogenous application also influencing fruit morphological traits. During grape berry development, calcium levels tend to stabilize after veraison, whereas potassium continues to accumulate until maturity. This indicates that calcium may play a predominant role during the early stages of development (Rogiers et al., 2006). Regarding fruit size, exogenous application of Abscisic Acid (ABA), a phytohormone that primarily functions as a growth inhibitor and stress signaling molecule in plants, has been shown to promote calcium accumulation in tomatoes and positively regulate developmental processes, thereby enhancing the fruit size (Tonetto De Freitas et al., 2014). In ‘Red Delicious’ apples, nano‐calcium treatment significantly increases fruit weight and size, which is attributed to calcium ions acting as cellular signaling co‐messengers that promote cell division and thereby enhance fruit development (Ranjbar et al., 2019). However, the efficacy of calcium treatments in fruit development varies significantly across species. For example, calcium treatments have been shown to inhibit fresh weight accumulation, delay ripening, and reduce fruit size in certain grape cultivars and cherries (Martins et al., 2021). Additionally, its regulatory effects on fruit size and morphology are less evident in blueberry (*Vaccinium corymbosum*) (Hirzel, 2023), and sweet pepper (*Capsicum annuum*) (Michałojć and Dzida, 2008). In developing systems such as pollen tubes, it both cross‐links pectin to provide structural support and acts as a signaling mediator through protein interactions and ion channel modulation (Jiang et al., 2005; Konrad et al., 2011; Rounds et al., 2011). This raises the question of whether calcium similarly regulates both structural integrity and signaling networks during fruit maturation to coordinate growth and ripening. This dual role is clearly manifested in the regulation of fruit epidermal quality in grape (Figure 2; Table 1). For example, it structurally promotes the deposition of pectin‐like materials in skin cell walls, thickening the peripheral pectin layer, and smoothing the fruit epidermis. Simultaneously, as a signaling molecule, calcium triggers the coordinated downregulation of genes related to pectin modification, including pectin methylesterase 1 (*PME1*), polygalacturonases 1/2 (*PG1/PG2*), and expansin 6 (*EXP6*), as well as cuticle biosynthesis genes such as ECERIFERUM 9 (*CER9*) and cytochrome P450 family 1 (*CYP15*). Together, these molecular changes reduce microcrack formation in the epidermis and enhance cell wall integrity, thereby improving cuticle smoothness and mechanical strength (Martins et al., 2020).

Regarding fruit shape, Quantitative Trait Loci (QTLs) identified in tomato are perhaps the best characterized for fruit species (Figure 2; Table 1). The ovate and sun loci influence elongated shapes, whereas the locule number (lc) and fasciated (fas) loci both modify locule number, and both influence the shape (Chen et al., 2023). Among these QTLs, *SUN* has been identified as the primary locus responsible for regulating the elongated shape of tomato fruit. In tomato, the *SUN* gene promotes longitudinal fruit growth by regulating polar cell division, thereby determining overall fruit shape. This gene encodes an SUN/IQ67‐DOMAIN (IQD) protein containing calmodulin‐binding motifs, indicating its potential role in modulating cytoskeletal dynamics and gene expression via calcium signaling pathways, which may directly influence fruit morphology (Abel et al., 2013; Clevenger et al., 2015; Bürstenbinder et al., 2017). Recent studies have demonstrated that the tomato protein SlIQD21a/SUN10 directly regulates fruit shape by interacting with the microtubule‐binding protein SlMAP70 to modulate microtubule dynamics (Bao et al., 2023). Evidence in melon (*Cucumis melo*) further supports IQD‐mediated development via calcium signaling, where the direct interaction between CmSUN23‐24 and CmCaM5 provides evidence that IQD proteins also regulate development through calcium signaling (Ma et al., 2022). Moreover, members of the IQD protein family have been demonstrated to play a significant role in determining fruit shape in wax gourd (*Benincasa hispida*), watermelon (*Citrullus lanatus*) (Dou et al., 2018), and grape, where its function is modulated by indole‐3‐acetic acid (IAA) levels (Zheng et al., 2022). Additional research has indicated that the OVATE Family Protein (OFP)‐TONNEAU1 Recruiting Motif (TRM) interaction may operate in coordination with other shape‐regulating proteins, such as IQD, to ensure the formation of consistent and reproducible organ shapes (Snouffer et al., 2020). While studies on SUN/IQD proteins have thoroughly detailed their impact on fruit shape, the direct connection between calcium signaling and these proteins has not been firmly established.

### Calcium and fruit color

Fruit color, as the most direct sensory attribute, plays a critical role in shaping consumer preferences and determining commercial value. During fruit development and ripening, the initial green coloration conferred by chlorophyll gradually transitions to species‐specific hues at maturity, a process mediated by distinct classes of secondary metabolites. These include carotenoids (yellow‐orange pigments) (Xue et al., 2024), anthocyanins (pH‐dependent red/blue/purple pigments) (Zhao et al., 2024), and betalains (stable red/yellow pigments) (Zhou et al., 2020). This chromatic transformation not only serves as a biological indicator of ripeness but also reflects the complex and dynamic regulatory networks that govern pigment metabolism. Despite accumulating evidence, the precise regulatory mechanisms by which calcium signaling influences fruit coloration remain incompletely understood. This section will focus on two major classes of pigments that directly determine fruit color, anthocyanins and carotenoids (Figure 2; Table 1).

Previous studies have elucidated the role of calcium in anthocyanin accumulation. Compared with normal apple peels, endogenous calcium‐deficient apple peels exhibit reduced anthocyanin content (Sun et al., 2021), whereas exogenous calcium application has been shown to increase anthocyanin levels in various fruits, including apple (Solhjoo et al., 2017), diploid woodland strawberry (*Fragaria vesca*) (Xu et al., 2014), grape (Yu et al., 2020b), “Shuguang” nectarines (*P. persica* var. *nucipersica*) (Zhu et al., 2023), and cherry (*Prunus avium*) (Michailidis et al., 2017). Although the precise mechanisms by which calcium regulates anthocyanin biosynthesis in fruits remain largely unclear, preliminary findings suggest its potential involvement in four pathways related to anthocyanin synthesis (Yu et al., 2020b).

First, calcium enhances the transcription of key genes involved in the anthocyanin biosynthesis pathway, including flavanone 3‐hydroxylase (*F3H*), dihydroflavonol 4‐reductase (*DFR*), anthocyanidin synthase (*ANS1*), *UGT1*, and UDP‐glucose: flavonoid 3‐O‐glucosyltransferase (*UFGT*), thereby promoting anthocyanin production. In the grape, calcium application upregulates the expression of anthocyanin biosynthesis‐related genes, such as *VvDFR* and *VvUFGT*, including transporter genes Anthocyanin MATE 1 (*VvAM1*) and ATP‐Binding Cassette transporter C1 (*VvABCC1*) (Martins et al., 2018). Similarly, nano‐calcium application in “Shuguang” nectarines significantly increases the expression of *PpUFGT* in the fruit peel (Zhu et al., 2023). In the diploid woodland strawberry, calcium promotes the expression of structural genes, including *F3H1*, *DFR2*, *ANS1*, and *UGT1*. Further studies have demonstrated that the activity of FvUGT1 is inhibited by its substrate, anthocyanidin; however, the binding of CaM complexes to FvUGT1 mitigates this substrate‐induced inhibition, thereby promoting enhanced anthocyanin accumulation (Peng et al., 2016). Second, calcium modulates transcription factors to amplify the anthocyanin biosynthesis pathway. Within the MYB‐bHLH‐WD40 (MBW) complex, the MYB transcription factor directly binds to promoters of anthocyanin biosynthetic genes to activate their transcription (Hichri et al., 2011). In pear (*Pyrus pyrifolia*) cultivars exhibiting marked differences in coloration, the transcription factors *PbMYB10* and *PbWD40* display distinct expression patterns (Feng et al., 2010). Similarly, calcium application in “Shuguang” nectarines significantly upregulates the expression of *PpMYB10.1*, potentially through a CaM‐mediated signaling mechanism (Zhu et al., 2023). Furthermore, in grapevine, calcium signaling acts synergistically with the jasmonic acid (JA) pathway to co‐activate MYB‐type transcription factors. Calcium treatment also broadly upregulates other anthocyanin‐related factors, such as *bZIP*, *bHLH*, and *NAC* (Yu et al., 2020b). In strawberry, Ca^2+^‐CaM complexes activate downstream kinases that phosphorylate and enhance the transcriptional activity of MYB10 (Peng et al., 2016). Collectively, these examples underscore a central mechanism by which calcium signaling regulates anthocyanin biosynthesis through the multi‐layered potentiation of transcription factor function. Third, calcium may indirectly promote anthocyanin accumulation by increasing soluble sugar content (SSC). Calcium has been demonstrated to participate in sugar‐induced anthocyanin biosynthesis, as evidenced by studies using calcium and CaM antagonists in grape cell cultures (Vitrac et al., 2000). In grape berries, exogenous calcium elevates fruit skin sugar and anthocyanin content, likely by upregulating sugar transporter genes such as *SUT2* and *HT* and thereby activating the anthocyanin synthesis pathway (Yu et al., 2020b). In Arabidopsis, calcium acts as a key mediator linking sugar signaling to anthocyanin synthesis. Changes in intracellular calcium levels regulate the expression of the sucrose transporter *SUC1*, thereby influencing sugar uptake. The resulting increase in sugar content further promotes anthocyanin accumulation by activating the transcription factor PAP1 and upregulating anthocyanin biosynthetic genes (Shin et al., 2013). Fourth, calcium signaling integrates hormonal and other signaling pathways to influence fruit coloration. Calcium promotes the synthesis of endogenous JA and ethylene (ETH), which in turn activate anthocyanin‐related transcription factors such as *MYB* or enhance sugar metabolism, thereby promoting anthocyanin accumulation (Yu et al., 2020b). However, in grape, calcium partially inhibits methyl jasmonate (MeJA) signaling when applied simultaneously, thereby attenuating MeJA's stimulatory effect on anthocyanin biosynthesis (Martins et al., 2018). Moreover, ultraviolet‐B (UV‐B) irradiation can counteract the suppressive effect of calcium by upregulating *miR3627/4376* (Sunitha et al., 2019), which antagonizes calcium effector proteins, while also inducing JA signaling to indirectly promote pigment accumulation (Martins et al., 2018; Zhu et al., 2023). Under cold stress conditions in the pepper, CaCIPK13 interacts with CBLs to activate calcium signaling. This signaling cascade enhances antioxidant enzyme activity and upregulates the anthocyanin biosynthesis pathway, thereby improving cold tolerance and promoting anthocyanin accumulation (Ma et al., 2021). Notably, the red striping observed in “Red Zaosu” pears is attributed to calcium‐dependent anthocyanin accumulation in the vicinity of vascular tissues, which is triggered by the upregulation of gibberellin 2‐oxidase 8 (*PbGA2ox8*) (Zhai et al., 2019). Furthermore, genome‐wide association studies in pepper have identified single‐nucleotide polymorphisms associated with fruit coloration within genes encoding mitochondrial CAXs, suggesting that calcium homeostasis may indirectly influence plastid pigment deposition by regulating mitochondrial function (Ro et al., 2024).

Compared with studies on anthocyanins, those on the role of calcium in carotenoid metabolism are relatively limited. In shredded carrots (*Daucus carota*), calcium chloride (CaCl_2_) treatment induces the expression of the CaMs binding transcription factor‐*DcCAMTA4*, which directly binds to the promoters of carotenoid cleavage dioxygenases 1 and 4 (*DcCCD1/4*), thereby suppressing their transcription and delaying carotenoid degradation. In ‘Shuguang’ nectarines, nano‐calcium application upregulates key carotenogenic genes, including phytoene synthase (*PpPSY*), phytoene desaturase (*PpPDS*), zeta‐carotene desaturase (*PpZDS*), lycopene beta‐cyclase (*PpLCY‐B*), beta‐carotene hydroxylase (*PpCHY‐B*), and zeaxanthin epoxidase (*PpZEP*), leading to a doubling of beta‐carotene content in the pulp (Zhu et al., 2023). In sweet pepper, calcium application significantly increases carotenoid content, likely by promoting photosynthesis and pigment synthesis (Michałojć and Dzida, 2008). Similarly, in cauliflower (*Brassica oleracea* var. *botrytis*), combined lime and CaCl_2_ spraying elevates carotenoid levels, potentially through mechanisms such as membrane stabilization and activation of antioxidant systems (Pavithara et al., 2022). CaCO_3_ nanoparticles can enhance the content of carotenoids in tomato (Kaningini et al., 2025). In contrast, high calcium levels reduce carotenoid accumulation in tomato, possibly due to calcium‐mediated suppression of ethylene production and delayed ripening (Santhosh et al., 2023). These mechanisms may provide important insights into calcium‐mediated regulation of carotenoids in other horticultural fruits (Zhang et al., 2024b).

## CALCIUM‐DEPENDENT MODULATION OF FRUIT INTERNAL NUTRITIONAL PROPERTIES

### Calcium and flavor substances

**Figure 3:**
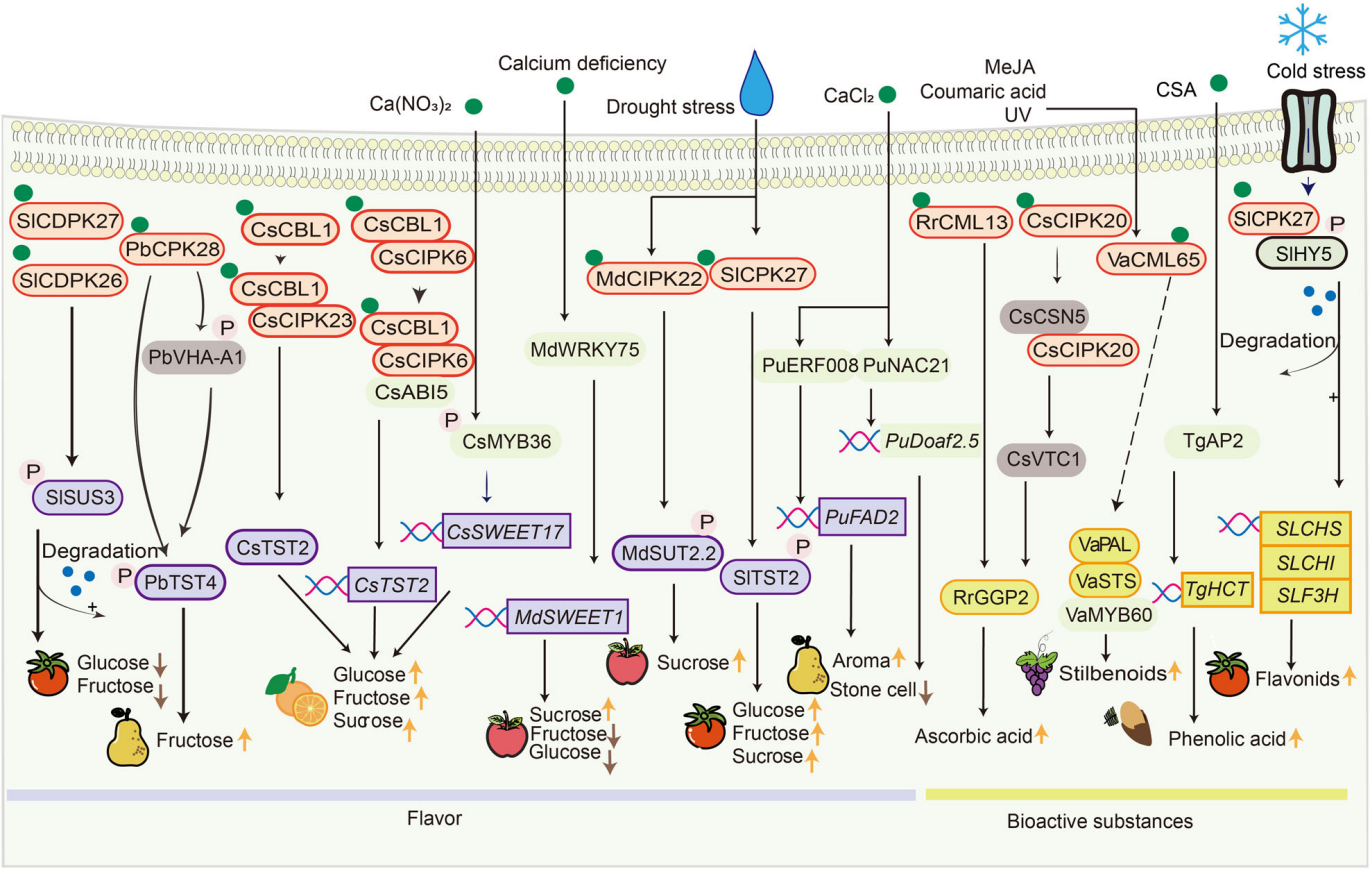
Calcium ions regulate the internal nutritional profiles of fruit Ca^2+^ ions can affect soluble solids content (SSC), as well as the contents of citric acid, aroma substances, and bioactive compounds in fruits. CSA, calcium‐sugar alcohol; CSN5, COP9 signalosome 5; FAD2, Fatty acid desaturase 2; GGP2, GDP‐L‐galactose phosphorylase 2; HCT, Hydroxycinnamoyl‐CoA: shikimate/quinate hydroxycinnamoyl‐transferase; MeJA, methyl jasmonate; SA, salicylic acid; SUS, Sucrose synthase; SUT, Sucrose transporter; TST, Tonoplast monosaccharide sugar transporter; UV, ultraviolet; VHA‐A1, Vacuolar hydrogen proton pump; VTC1, Vitamin C defective 1. Key symbols: Ub, ubiquitination; P, phosphorylation; upward arrow (↑), promotion or activation; downward arrow (↓), inhibition or suppression. Fruit drawings were drawn by the author using Adobe Illustrator, based on reference images available online.

The unique flavor profile of fruits is a critical determinant of consumer preferences and market value, arising from the synergistic interactions between volatile aroma compounds and non‐volatile components. From a chemical perspective, volatile flavor compounds mainly encompass aldehydes, esters, ketones, alcohols, terpenes, and sulfur‐containing compounds (El Hadi et al., 2013). The non‐volatile flavor constituents mainly comprise soluble sugars, such as sucrose, fructose, and glucose, as well as organic acids, including malic acid and citric acid (Klee and Tieman, 2018). Certain fruits also contain bitter compounds (Shang et al., 2014; Shui et al., 2019; Wu et al., 2022). Given their fundamental role in determining taste and the abundance of research available, this section will focus primarily on the metabolism and regulation of sugars and acids (Figure 3; Table 2).

**Table 2:** Key factors involved in calcium‐mediated regulation of internal nutritional profiles

Species	Components	Affected trait	Characteristic	Reference
*Solanum lycopersicum*	CDPK27/26‐SUS3	Sugar	CPK27/26 phosphorylates sucrose synthase to limit sugar accumulation.	(Zhang et al., 2024a)
*Pyrus bretschneideri*	CPK28‐TST4 VHA‐A1	Sugar	CPK28 phosphorylates TST4 and VHA‐A1 to boost sugar accumulation, and its promoter variant (SNP13T/C) affects fructose levels.	(Li et al., 2023)
*Citrus sinensis*	CBL1‐CIPK23‐TST2	Sugar	During citrus ripening, calcium‐sensing CBL1 activates CIPK23, which phosphorylates TST2 to boost sugar transport and sweetness.	(Li et al., 2024c)
*C. sinensis*	CIPK6‐ABI5‐TST2	Sugar	ABA kinase SRK2A and calcium kinase CIPK6 phosphorylate ABI5, activating *TST2* expression to promote sugar accumulation.	(Mao et al., 2025)
*Malus domestica*	WRKY75	Sugar	Calcium deficiency in apple upregulates WRKY75, which boosts *SWEET1* expression and sucrose accumulation.	(Sun et al., 2022)
*M. domestica*	CIPK22‐SUT2.2	Sugar	Under drought stress, CIPK22 phosphorylates SUT2.2 at Ser381, stabilizing it and boosting sugar transport into vacuoles.	(Ma et al., 2018)
*S. lycopersicum*	CPK27‐TST2	Sugar	CPK27 phosphorylates TST2 and enhances SSC under drought stress.	(Zhu et al., 2024a)
*Pyrus ussriensis*	ERF008‐FAD2	Aroma	CaCl_2_ triggers calcium signaling, leading to PuERF008 binding to the *FAD2* promoter, which accelerates aroma precursor synthesis and increases volatile aroma levels.	(Liu et al., 2024)
*P. ussriensis*	NAC21‐Dof2.5	Texture	Calcium suppresses *NAC21*, downregulating lignin genes and reducing the number of pear stone cells.	(Zhang et al., 2022)
*Rosa roxburghii*	CNGC12	AsA	Calcium activates CNGC12, promoting AsA accumulation.	(Wang et al., 2024)
*R. roxburghii*	CML13‐GGP2	AsA	Foliar application of Ca(CH_3_COO)_2_ activates CML13 and significantly enhances AsA synthesis.	(Yin et al., 2024)
*Camellia sinensis*	CIPK20‐CSN5‐VTC1	AsA	CIPK20 binds competitively to CSN5, blocks VTC1 degradation, and significantly enhances AsA synthesis.	(Di et al., 2024)
*Vitis vinifera*	CML65	Stilbene	Overexpression of *CML65* in grape callus cells activates *MYB60*, thereby promoting stilbene biosynthesis.	(Aleynova et al., 2022)
*Torreya grandis*	AP2‐HCT	Torreyic acid	Foliar application of calcium activates AP2, upregulates *HCT* expression, and promotes phenolic acid accumulation.	(Xie et al., 2025)
*S. lycopersicum*	CPK27‐HY5	Flavonid	Foliar spraying of CaCl₂ activates CPK27‐HY5 and boosts flavonoid accumulation.	(Lin et al., 2025)

Increasing studies have confirmed that exogenous calcium treatment can significantly influence soluble solid content (SSC) in various horticultural crops, including the litchi (Peng et al., 2022; Shi et al., 2024), melon, sweet cherry (Michailidis et al., 2022; Wu et al., 2023), apple (Ranjbar et al., 2020; Wang et al., 2022), grape (Zhu et al., 2019), “Nanguo” pears (Zhang et al., 2019a), and sweet pepper (Michałojć and Dzida, 2008). Similarly, endogenous calcium deficiency adversely affects sugar metabolism in apple, and the abnormal upregulation of *WRKY* transcription factors leads to aberrant sucrose accumulation, accompanied by significant reductions in glucose and fructose levels. This phenomenon not only reduces fruit sweetness but may also induce physiological disorders such as bitter pit (Sun et al., 2022). Molecular biological evidence further demonstrates that calcium signaling pathways directly participate in regulating SSC in fruits, with its regulatory mechanisms primarily involving two key aspects: sugar metabolic pathways and sugar transport/storage pathways.

In sugar metabolism, sucrose serves as the primary photosynthetic product in higher plants. The dominant form of sugar accumulation in fruits is a crucial source of glucose and fructose. Key enzymes governing sucrose metabolism include invertases (INV), comprising acid invertase (AINV) and neutral invertase (A/NINV), along with sucrose phosphate synthase (SPS) and the bidirectional enzyme sucrose synthase (SS), which mediates both synthetic (SS‐s) and cleavage (SS‐c) activities. In “Shuguang” nectarines, nano‐Ca upregulates sucrose synthase 1 and 3 (*PpSUS1*, *PpSUS3*) expression in leaves by calmodulin, which is the central calcium sensor that transduces signals to downstream transcriptional regulators (Zhu et al., 2023). In grape, calcium enhances sucrose cleavage into glucose and fructose by modulating the activity of sucrose‐metabolizing enzymes and likely interfaces with hormonal and phosphorylation‐based regulation, potentially via CDPKs or CBL‐CIPK, to shift the sucrose cleavage/synthesis balance (Li et al., 2024a). In apples, composite calcium fertilizers activate SPS and SS‐s, resulting in increased levels of sucrose and sorbitol (Wang et al., 2022). In “Red Fuji” apples, nano‐calcium application was found to delay fruit ripening, leading to increased starch content and decreased total soluble solids (TSS) (Ranjbar et al., 2019). Concurrently, suppressed respiration rates further minimized sugar consumption. Furthermore, in “Feizixiao” litchies, calcium‐magnesium treatment optimized energy use efficiency by balancing the cytochrome pathway (CP) and alternative pathway (AP) metabolic pathways, reducing wasteful substrate utilization and mitigating sugar decline (Shi et al., 2024). However, excessive calcium application may produce counterproductive effects by downregulating key sugar metabolism genes, ultimately leading to reduced SSC (Shui et al., 2022). At the molecular level, studies in tomato have revealed that SlCDPK27 and SlCDPK26 phosphorylate SlSUS3, promoting its degradation and consequently suppressing sucrose biosynthesis (Zhang et al., 2024a).

In terms of sugar transport and storage, these processes heavily rely on the regulation of source‐sink relationships. The “source” primarily refers to photosynthetic leaf tissues, which synthesize and export photoassimilates such as sucrose. The “sink” denotes tissues that receive, utilize, or store these assimilates, with fruits being a classic sink organ. Sucrose produced in the source leaves is transported over long distances via the phloem to sink organs such as fruits. Within sink tissues, the vacuole, as a crucial subcellular storage compartment, plays a central role in the storage and accumulation of sugars, acids, and other flavor compounds (Liu et al., 2023b). Based on previous reports, calcium treatment in “Shuguang” nectarine induces the expression of phloem‐specific sucrose transporter genes sucrose transporter 2 and 4 (*PpSUT2*, *PpSUT4*), promoting photoassimilate translocation from source leaves to sink fruits. In apple, phosphorylation of MdSUT2.2 by MdCIPK22 enhances its stability and promotes sugar accumulation under drought stress (Ma et al., 2018). Calcium application in pepper also enhances the transcription of hexose transporters (*HT*), sugar transporter Early Responsive to Dehydration 6 (*ERD6‐like*), and the bidirectional sugar will eventually be exported transporter 15 (*SWEET15*), promoting the transport of glucose and fructose into vacuoles, thereby increasing the content of SSC (Yu et al., 2020b). When it comes to sugar storage in vacuoles, research on TST proteins is relatively extensive. In tomato, CPK27 phosphorylates TST2, thereby enhancing SSC under drought stress (Zhu et al.,2024a). During sweet orange (*Citrus sinensis*) maturation, the CBL1 and CIPK23 signaling module phosphorylates the vacuolar sugar transporter TST2 to facilitate sugar accumulation in vacuoles (Li et al., 2024c), and a regulatory module consisting of CsSRK2A/CsCIPK6‐CsABI5‐CsTST2 integrates ABA and calcium signaling to enhance vacuolar sugar accumulation, ultimately boosting fruit sweetness (Mao et al., 2025). In pear, a single‐nucleotide polymorphism (SNP) variation in the promoter region of *PbCPK28* is associated with fructose content. PbCPK28 not only interacts with the vacuolar proton pump PbVHA‐A1, indirectly providing proton motive force for PbTST4 to enhance fructose accumulation, but also directly increases PbTST4's transport activity (Li et al., 2023).

According to multiple research studies, calcium treatment exerts complex and bidirectional effects on fruit acidity, with the specific outcome, either increasing or decreasing acidity, dependent on fruit species, developmental stage, application method, and the type of organic acid involved (Figure 3; Table 2). An acid‐enhancing effect has been observed in some fruits. For instance, in apples, calcium treatment inhibits the decline in titratable acidity and malic acid content, while increasing the concentrations of succinate and oxalate (Han et al., 2021; Angmo et al., 2022). In grapes, Pro‐Ca treatment enhances the accumulation of tartaric acid, malic acid, citric acid, and oxalic acid, particularly during the ripening stage (Li et al., 2024a). In sweet cherry, calcium spraying also significantly elevates the levels of malic acid, citric acid, fumaric acid, and quinic acid (Nagy et al., 2008). On the other hand, calcium treatment can also exhibit acid‐reducing effects under certain conditions. For example, it reduces the percentage of titratable acidity in tomato (Dong et al., 2005) and decreases titratable acidity or specific organic acids such as glyceric and threonic acids in some apples and sweet cherries (Michailidis et al., 2022). Furthermore, in ‘Shine Muscat’ grapes at the veraison stage, calcium treatment promotes the degradation of malic and citric acids (Wang et al., 2021a). These reports indicate that the regulation of fruit acid metabolism by calcium is highly context‐dependent, and its overall effect should be interpreted with consideration of the physiological stage and species‐specific characteristics.

Calcium controls fruit organic acid metabolism through multiple mechanisms (Table 2). At the enzymatic activity level, calcium treatment can significantly regulate the activity of key metabolic enzymes. It generally suppresses acid synthesis by inhibiting key enzymes such as phosphoenolpyruvate carboxylase (PEPC) and citrate synthase (CS) (Jaime‐Guerrero et al., 2024), while promoting acid degradation via enzymes like malate dehydrogenase (MDH) or citrate lyase (CL), as observed during grape veraison (Wang et al., 2021a). In pear, calcium may enhance citrate degradation by modulating CS activity (Sha et al., 2018). By contrast, in apple, postharvest calcium treatment increases the activity of cytosolic NAD‐dependent malate dehydrogenase (cyNAD‐MDH) and PEPC, while inhibiting cytosolic NADP‐dependent malic enzyme (cyNADP‐ME) and phosphoenolpyruvate carboxykinase (PEPCK) to suppress the decline in malate content (Han et al., 2021), reflecting a species‐specific regulatory pattern. In grapes, Pro‐Ca application modulates the activities of key enzymes, including CS, PEPC, and NADP‐ME, thereby promoting the accumulation of tartaric acid, malic acid, citric acid, and oxalic acid (Li et al., 2024a). At the structural level, calcium is capable of forming stable complexes with organic acids. The binding of calcium to organic acids reduces the concentration of free organic acids. In grapes, calcium may react with malic and tartaric acids to form insoluble salts, thereby influencing titratable acidity (Possner and Kliewer, 2015). In tomato, calcium is known to form complexes with oxalate and citrate in the cell wall (Paiva et al., 2023). The formation of these calcium‐organic acid complexes affects the spatial distribution and bioavailability of organic acids, as the oxalic acid undergoes biomineralization to form calcium oxalate crystals within idioblasts, a process that sequesters oxalic acid in specific cellular compartments (Possner and Kliewer, 2015). Calcium also regulates organic acid content by modulating vacuolar transport and interacting with key mineral ions. At the vacuolar membrane, it inhibits the activity of proton pumps, including Proton pump ATPase (H^+^‐ATPase) and vacuolar pyrophosphatase (V‐PPase), reducing the active transport of organic acids into the vacuole (Liao et al., 2022). Calcium also interacts synergistically and antagonistically with other ions, as it influences magnesium content, which is an essential activator of V‐PPase, thereby affecting pump activity and acid metabolism. Additionally, calcium can lower fruit acidity by competing with potassium for uptake or inhibiting its transport into the fruit, counteracting potassium's promotion of organic acid accumulation (Liao et al., 2022).

Besides, exogenous calcium treatment is an effective strategy for improving fruit aroma quality through synergistic physical and biochemical mechanisms. Physically, calcium modifies the structure of cuticular wax, promoting wax shedding and enlarging intercellular spaces and lenticel apertures, thereby reducing the diffusion resistance of volatile compounds and enhancing aroma release (Wei et al., 2021). Biochemically, calcium acts as a signaling molecule that activates key transcriptional modules, such as PuERF008‐PuFAD2, significantly increasing the activity of enzymes including lipooxygenase (LOX), alcohol dehydrogenase (ADH), and pyruvate decarboxylase (PDC), and upregulating fatty acid desaturase genes to promote the synthesis of ester aroma precursors and final compounds (Liu et al., 2024). Calcium also elevates β‐glucosidase activity, facilitating the hydrolysis of bound aroma compounds into free volatiles (Wei et al., 2017). Additionally, it modulates endogenous hormone balance and sugar‐acid metabolism to supply substrates and energy for aroma synthesis. For example, Pro‐Ca treatment reduces ABA levels while increasing Zeatin (ZT), Gibberellin A_3_ (GA_3_), and IAA levels, promoting ripening and creating favorable physiological conditions for aroma production in grapes. It also enhances the activity of sugar‐metabolism enzymes (e.g., SuSy‐s, SPS, AINV) and organic acid‐metabolism enzymes (e.g., CS, PEPC), thereby raising the contents of soluble sugars and organic acids, which provide richer precursor pools and energy for aroma biosynthesis (Li et al., 2024a). However, the specific underlying mechanisms require further investigation.

### Calcium and bioactive substances

During fruit maturation, calcium exerts a significant regulatory influence on the synthesis and accumulation of bioactive compounds, including polyphenols and flavonoids, via secondary metabolic pathways (Kiselev and Dubrovina, 2025). These compounds play diverse roles in plants, functioning as defense agents against pathogen invasion, structural components for cell wall reinforcement, signaling molecules regulating growth processes, and antioxidants contributing to cellular homeostasis and enhanced stress tolerance (Erb and Kliebenstein, 2020). From a human health perspective, these bioactive compounds confer numerous benefits, including potent anti‐inflammatory effects, modulation of glucose and lipid metabolism, and potential anti‐carcinogenic properties, all of which contribute significantly to overall health and well‐being (Li et al., 2025a).

A body of evidence indicates that the application of exogenous calcium serves as an effective strategy to enhance the biosynthesis and accumulation of bioactive compounds in horticultural crops. Studies have demonstrated that CaCO_3_ nanoparticles (NPs) can enhance the content of total flavonoids, ascorbic acid (AsA), and lycopene in the ripe fruits of tomato cultivars “Heinz‐1370” and “Money‐maker” (Kaningini et al., 2025; Motlhalamme et al., 2025). CaCl_2_ treatment promotes the accumulation of total flavonoids and capsaicin in the pepper (Montri et al., 2024). Furthermore, the combined application of exogenous brassinosteroids (BR) and CaCl_2_ has been found to mitigate the decline of total phenols, flavonoids, and AsA in jujube (*Ziziphus ea*) (Ban et al., 2024). In peach, CaCl_2_ treatment was reported to increase the levels of pathogenesis‐related proteins, flavonoids, total phenolics, and anthocyanins by activating phenylpropanoid metabolism (Li et al., 2025b). In grape callus cells, overexpression of *CML65* callus cells activates *MYB60*, thereby promoting stilbene biosynthesis (Aleynova et al., 2022). Notably, a recent study uncovered a novel regulatory mechanism by which calcium‐sugar alcohol (CSA) fertilizer enhances phenolic acid biosynthesis in “Xiangfei” nuts (*Torreya grandis*) through the Ca^2+^‐AP2‐HCT module (Xie et al., 2025).

Calcium has been widely studied for its role in enhancing various phytochemicals, such as capsaicin, lycopene, and phenolics. Especially, its most extensively investigated effect has been on the regulation of AsA, which is an essential nutrient known for its antioxidant properties and its ability to enhance immune function. Research has demonstrated that calcium treatments can significantly increase AsA content in a variety of fruits, including “Shuguang” nectarines (Zhu et al., 2023), “nanfeng tangerines” (Chen et al., 2024), apple (Reitz and Mitcham, 2025), eggplant (Ban et al., 2021), apricot (Moradinezhad and Dorostkar, 2021), sweet cherry (Michailidis et al., 2017), tea plant (*Camellia sinensis*) (Di et al., 2024), and Roxburgh rose (*Rosa roxburghii*). The proposed mechanisms for this enhancement encompass multiple synergistic pathways, including the delay of ripening and senescence through ethylene suppression, the reinforcement of the antioxidant system to mitigate AsA oxidation, the stabilization of cellular structures to prevent AsA loss, and the promotion of sugar metabolism to supply biosynthetic precursors. Especially, Roxburgh rose, a perennial shrub native to calcium‐rich karst areas in southwestern China, is particularly notable due to its remarkably high content of AsA, triterpenoids, and flavonoids, which have been associated with anti‐aging and anticancer properties (Ojo et al., 2022). Ca^2+^ has been shown to promote the expression of genes involved in AsA biosynthesis, such as galactonolactone dehydrogenase and GDP‐L‐galactose phosphorylase (GGP), as well as those involved in its regeneration pathway, including dehydroascorbate reductase (DHAR) and monodehydroascorbate reductase (MDHAR) (Yin et al., 2024). Exogenous calcium promotes the accumulation of AsA in Roxburgh rose fruits by initiating nuclear interactions between CML13 and GGP2, which is a key enzyme involved in AsA biosynthesis in plants (Yin et al., 2024). Moreover, calcium synergistically enhances the biosynthesis of AsA, flavonoids, and triterpenoids in these fruits, with CNGC12 serving as a central regulatory component in this process (Wang et al., 2024).

## CALCIUM AND PHYSIOLOGICAL DISORDERS

**Figure 4:**
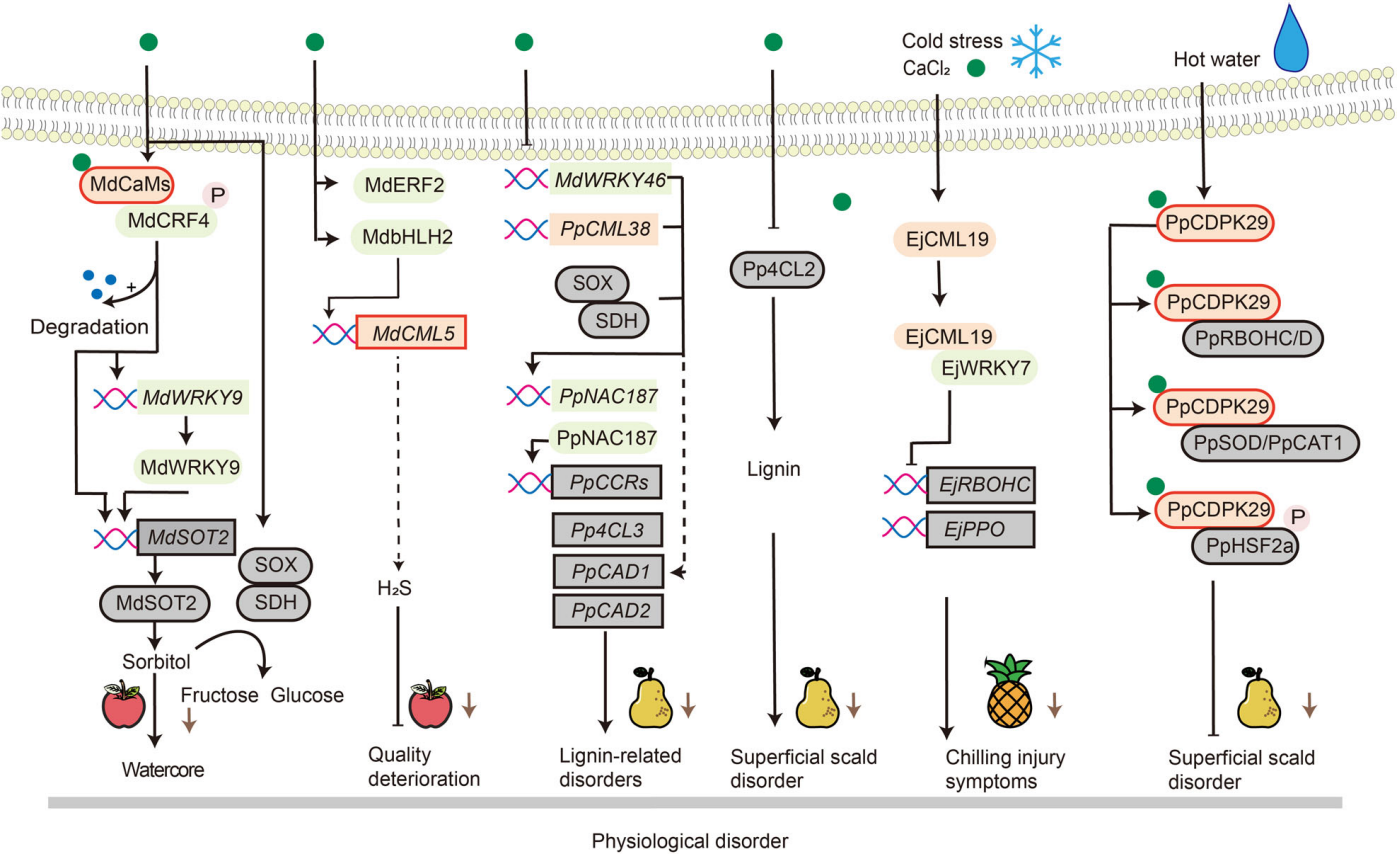
Calcium ions alleviate physiological disorders in fruit A range of Ca^2+^‐related molecular components, including the critical genes *CAX*, *CML*s, and *CDPK*s, regulate fundamental aspects of fruit physiology, from sorbitol metabolism and lignin biosynthesis to H₂S/reactive oxygen species (ROS) signaling and Ca^2+^ homeostasis. The regulation of these pathways controls the development of physiological disorders, such as watercore, superficial scald, chilling injury, and fruit cracking. 4CL3, 4‐Coumarate‐CoA ligase 3; CAD, Cinnamyl alcohol dehydrogenase; CAT, Catalase; CCR, Cinnamoyl‐CoA reductase; HSF, Heat‐shock factor; PPO, Polyphenol oxidase; RBOH, Respiratory burst oxidase homolog; SDH, Sorbitol dehydrogenase; SOD, Superoxide dismutase; SOT, Sorbitol transporter; SOX, Sorbitol oxidase. Key symbols: Ub, ubiquitination; P, phosphorylation; upward arrow (↑), promotion/activation; downward arrow (↓), inhibition/suppression. Fruit drawings were drawn by the author using Adobe Illustrator, based on reference images available online.

Physiological disorders refer to functional impairments in plant tissues that result from abiotic stress factors, such as nutrient imbalances, water stress, extreme temperatures, or metabolic dysfunctions. In contrast to biotic stresses, such as infections caused by pathogens, physiological disorders generally present as localized and irreversible damage, such as browning, pitting, cracking, or necrosis. These symptoms often appear subtly and may remain latent until postharvest storage, thereby negatively affecting fruit storability, yield, quality, and overall market value (Figure 4; Table 3).

**Table 3:** Key factors involved in calcium‐mediated regulation of physiological disorders

Species	Components	Affected trait	Characteristic	Reference
*Malus domestica*	crf4‐wrky9	Watercore	Ca^2+^ alleviates apple watercore by inducing ubiquitination and degradation of MdCRF4, thereby suppressing the MdWRKY9‐MdSOT2 transcriptional cascade and reducing sorbitol accumulation.	(Li et al., 2024b)
*Mangifera indica*	ACA CAX	Spongy tissue	Calcium deficiency disrupts pectin metabolism and promotes vacuolar calcium sequestration, resulting in abnormal cell walls.	(Ma et al., 2023)
*M. domestica*	CAX5/11	Bitter pit	High CAX expression may lead to excessive calcium influx into vacuoles, reducing cytosolic calcium availability and contributing to bitter pit.	(Liu et al., 2021)
*Pyrus bretschneideri*	CMLs	Superficial scald	Postharvest fruit immersion in 2% CaCl_2_ for 15 min significantly reduces superficial scald of PE‐bagged pear fruit.	(Li et al., 2020)
*P. bretschneideri*	CML38/WRKY46‐NAC187‐CCR	Lignin‐related physiological	Postharvest fruit immersion in 2% CaCl_2_ inhibits the CML38‐mediated regulatory module, suppresses lignin accumulation, and thereby reduces related physiological disorders.	(Cheng et al., 2025)
*P. bretschneideri*	4CL2	Superficial scald	Postharvest fruit immersion in 2% CaCl_2_ for 15 min downregulates *4CL2*, reduces pear fruit lignin, and alleviates superficial scald during storage.	(Li et al., 2022a)
*Eriobotrya japonica*	CML19‐WRKY7	Chilling injury	Postharvest fruit immersion in 1% CaCl_2_ inhibits browning gene expression, reduces phenolic oxidation and membrane lipid degradation, and alleviates chilling injury in cold‐stored fruit.	(Hou et al., 2023)
*Ananas comosus*	CAX2	Flesh translucency	The dysfunction of calcium transporter CAX2 causes low fruit calcium and flesh translucency.	(Shu et al., 2025)
*Pyrus prifolia*	CML11	Fruit cracking	CML11 may be involved in flesh development and play an important role in responding to biotic stress after fruit cracking.	(Fan et al., 2023)
*Prunus persica*	CDPK29	Chilling injury	Hot water treatment alleviates postharvest chilling injury in peach fruit by coordinating Ca^2+^‐ROS signaling and the HSF‐HSP pathway via PpCDPK29.	(Zhao et al., 2025)

Calcium, an essential plant nutrient, maintains fruit physiological homeostasis through three core mechanisms. First, in structural role, it stabilizes cell wall integrity via pectin‐Ca^2+^ crosslinking and maintains membrane structure (Rosenberger et al., 2004). Second, it modulates redox balance by regulating antioxidant enzyme activity and reactive oxygen species (ROS) metabolism (Santhosh et al., 2023). Finally, it coordinates stress responses by influencing key signaling pathways, particularly those involved in ETH biosynthesis and stress‐related signal transduction cascades. However, calcium uptake in fruits is primarily dependent on transpiration‐driven xylem transport, and its capacity for remobilization during ripening is limited. During the phase of fruit expansion, competition between carbon and calcium allocation, as well as environmental stresses such as heat or drought, may result in localized calcium deficiency (Thor and Peiter, 2014). Such an imbalance in Ca^2+^ levels predisposes fruits to common physiological disorders, such as bitter pit, water core, and fruit cracking, and accelerates postharvest browning and softening. Moreover, numerous studies have shown that excessive CAX activity depletes calcium, an essential cellular nutrient, resulting in localized calcium deficiency. This section systematically examines the crucial role of calcium in the development of fruit physiological disorders.

### Calcium and fruit cracking

Fruit cracking is a prevalent phenomenon that results in substantial economic losses. Several factors influence fruit cracking, including cell wall composition, soluble solid content, hormonal regulation, and mineral nutrition (Shi et al., 2022). Among these, calcium deficiency has been identified as a critical factor contributing to fruit cracking in horticultural crops. In grape (Yu et al., 2020a), lemon (Devi et al., 2018), litchi (Martínez Bolaños et al., 2017), sweet cherry (Michailidis et al., 2020; Michailidis et al., 2021; Michailidis et al., 2022), and pomegranate (Davarpanah et al., 2018), insufficient calcium compromises the flexibility of cell walls and the structural integrity of cell membranes. Under conditions of abnormal internal pressure caused by water uptake or metabolic processes, this deficiency ultimately leads to epidermal rupture. Calcium supplementation has been shown to mitigate this issue to some extent (Khadivi‐Khub, 2014; Fischer et al., 2021). In sweet cherry, transcriptomic analysis shows that calcium treatment reduces respiration rate and alters metabolite levels, thereby decreasing fruit cracking (Michailidis et al., 2020). Additionally, spraying calcium during the dormancy period can effectively increase the calcium content in sweet cherry fruits and reduce the fruit cracking rate (Michailidis et al., 2021). Calcium may enhance fruit strength by strengthening the cell wall and epidermal structure, as well as by reducing the water permeability of the fruit skin (Measham et al., 2013). In pear, the calcium content in both peel and flesh of cracked fruits is markedly lower than that in healthy fruits. Notably, the expression level of *PpCML11* is elevated in the peel and flesh of cracked fruits compared to non‐cracked fruits. This disruption in the calcium signaling pathway may indirectly interfere with the coordinated regulation of fruit development (Fan et al., 2023). Moreover, ETH signaling may exacerbate fruit cracking by impairing calcium transport. Additionally, abiotic stresses that induce imbalances in mineral absorption, such as competitive interactions between potassium or magnesium and calcium, further compromise calcium homeostasis, thereby promoting fruit cracking (Lee et al., 2016; Fernandez‐Munoz et al., 2022). Besides, calcium stabilizes cell walls by cross‐linking pectin, a process involving the GAUT (galacturonosyltransferase) complex, which affects fruit cracking and softening (Atmodjo et al., 2013).

### Calcium and fruit softening

Beyond its visible manifestation in fruit cracking, cell wall degradation has been recognized as a pivotal factor in fruit firmness and postharvest fruit softening, with both processes sharing common pathways of cellular structure destabilization. Studies demonstrate a positive correlation between fruit firmness and calcium content in both cell walls and membrane systems. For instance, high‐firmness blueberries exhibit significantly higher calcium concentrations in their cell walls compared to softened fruits (Olmedo et al., 2021). Exogenous calcium treatment has been proven effective in delaying softening across various fruits, including apple (Rabiei et al., 2011; Xu et al., 2023), citrus (Chen et al., 2024), persimmon (Liu et al., 2023a), and plum (Koricanac et al., 2021). Molecular evidence further demonstrates that transgenic tomatoes overexpressing the Arabidopsis vacuolar calcium transporter gene *AtCAX4* exhibit significantly increased calcium accumulation, improved firmness, and prolonged storage life (Park et al., 2005), highlighting the critical role of calcium homeostasis in sustaining postharvest fruit quality.

### Calcium and superficial scald

To ensure the viability of long‐distance transport and trade, harvested fruits are typically stored at low temperatures. However, such storage may induce undesirable physiological changes that negatively affect the fruit's commercial value and market price. Superficial scald, a classic cold‐induced disorder in pears characterized by skin browning and necrosis, significantly compromises fruit marketability. Studies on ‘Chili’ pears have linked scald development to the upregulation of calcium‐signaling genes, specifically *PpCMLs* and *PbCNGC1* (Li et al., 2020). This gene expression pattern mirrors the stress‐induced upregulation of *AtCML9* in Arabidopsis under salt, cold, or drought stress, suggesting that chilling stress disrupts calcium homeostasis to initiate scald. Supporting this, exogenous CaCl_2_ application effectively mitigates scald symptoms. Mechanistically, calcium acts by suppressing lignin biosynthesis, downregulating the key gene *Pp4CL2* (Li et al., 2022a) and interfering with the CML38/WRYK46‐NAC187‐CCR transcriptional cascade (Cheng et al., 2025), thereby ultimately inhibiting lignification. Similarly, calcium inhibits the expression of lignin synthesis genes through the PuNAC21‐PuDof2.5 transcriptional module, reducing the formation of stone cells in pears (Zhang et al., 2025a). These findings elucidate a calcium‐mediated regulatory pathway against chilling injury and offer novel strategies for managing disorders related to aberrant lignification.

### Calcium and postharvest browning

Postharvest browning constitutes a major challenge to fruit quality, arising from complex interactions among environmental stress, pathogen infection, and endogenous metabolic imbalances. The underlying mechanisms primarily involve three interconnected pathways. The primary and most direct mechanism involves a disruption in phenolic metabolism, in which phenylalanine ammonia‐lyase (PAL), the rate‐limiting enzyme responsible for the synthesis of phenolic precursors in the phenylpropanoid pathway, initiates the biosynthesis of phenolic compounds, followed by the oxidation of these compounds into brown quinone pigments catalyzed by polyphenol oxidase (PPO) and peroxidase (POD) (Youryon et al., 2018; Ban et al., 2021; Mishra et al., 2022; Li et al., 2025b). For instance, in eggplants (*Solanum melongena*), CaCl_2_ application maintains higher phenolic content and suppresses PPO activity (Ban et al., 2021). Organic derivatives like calcium ascorbate (CaASC) and calcium gluconate (CaGlu) offer superior permeability, enabling more effective key enzyme inhibition and reduced browning (Youryon et al., 2018; Allegra et al., 2022; Mishra et al., 2022). The second pathway involves oxidative damage triggered by ROS, which accumulates directly by oxidizing phenolic compounds. Concurrently, membrane damage instigates lipid peroxidation and electrolyte leakage, thereby forming a vicious cycle that perpetuates ROS production (Ranjbar et al., 2018; Zhang et al., 2019b; Zhang et al., 2022; Peng et al., 2024). Calcium treatment counteracts this oxidative burst by augmenting the ROS‐scavenging system. In eggplants, calcium treatment enhances the activity of ROS‐scavenging enzymes, leading to reduced levels of H_2_O_2_ and O_2_^−^ compared to water controls, along with increased accumulation of non‐enzymatic antioxidants like AsA (Ban et al., 2021). Similarly, a pre‐storage application of a CaCl_2_‐citric acid solution mitigates chilling injury‐induced browning through the upregulation of antioxidant genes, including *catalase* (*CAT*), superoxide dismutase (*SOD*), and *glutathione reductase* (*GR*) in peach (Mishra et al., 2022). A third pathway involves calcium signaling and hormonal networks, which indirectly regulate browning through the modulation of antioxidant enzyme activity and ROS metabolism (Peng et al., 2024). For example, calcium signaling can reduce ROS accumulation by enhancing antioxidant enzyme activity and may interact with JA and IAA signaling pathways to jointly regulate browning processes. Moreover, calcium influences pivotal transcription factors, including WRKY and NAC families, thereby indirectly orchestrating the browning response (Wang et al., 2021b; Hou et al., 2023; Li et al., 2025b). A prominent example is the calcium‐regulated EjCML19‐EjWRKY7 transcriptional complex, which suppresses *EjPPO* expression. The above mechanisms offer promising targets for developing precise anti‐browning strategies (Hou et al., 2023).

### Calcium and other fruit‐specific physiological disorders

Beyond common postharvest disorders, calcium deficiency is also a key causal factor in several fruit‐specific physiological disorders, including watercore, flesh translucency, bitter pit, blossom‐end rot, and spongy tissue. Watercore, a common physiological disease in *Rosaceae* fruits, such as apples and Chinese white pears (Zupan et al., 2015), usually occurs during the fruit ripening period. Previous studies have demonstrated that Ca^2+^ plays a role in the development of watercore in apples. The calcium content in watercore‐affected tissue is significantly lower than in non‐watercore tissue (Yang et al., 2023). At the molecular level, apple watercore is regulated by calcium signaling, which promotes the ubiquitination‐mediated degradation of the transcription factor MdCRF4. This process suppresses the expression of the sorbitol transporter 2 (*MdSOT2*), thereby reducing sorbitol accumulation and alleviating watercore symptoms (Li et al., 2024b). Flesh translucency is associated with calcium deficiency. In pineapples (*Ananas comosus*), the *AcoCAX2* gene plays a key role in the transport of Ca^2+^ from the stem phloem to the developing fruits. Mutations in *AcoCAX2* have been shown to reduce calcium accumulation in fruits, which correlates with a significantly increased incidence of flesh translucency compared with wild‐type controls (Shu et al., 2025).

Moreover, bitter pit (BP) in apples is recognized as a physiological disorder caused by Ca^2+^ deficiency, which compromises fruit quality and leads to significant economic losses. The application of calcium in combination with a surfactant has been shown to elevate apoplastic calcium concentrations and reduce the incidence of BP compared to fruits treated with water alone (Reitz and Mitcham, 2025). Furthermore, a study has reported an intriguing finding that ABA not only mitigates BP by enhancing calcium accumulation in apples but also downregulates the expression of the *CAX* gene (Falchi et al., 2017). Notably, research indicates that BP primarily arises from abnormal local calcium distribution rather than an overall deficiency in total calcium levels (De Freitas et al., 2010; Torres et al., 2024). Further investigations reveal that in BP‐affected fruits, *MdCAX11* exhibits higher expression in the vacuolar membrane at the calyx‐end compared to the peduncle‐end. This differential expression pattern contributes to reduced levels of both cell wall‐bound calcium and cytosolic‐free calcium, ultimately leading to plasma membrane destabilization and the manifestation of bitter pit symptoms (Liu et al., 2021). Similarly, tomato blossom‐end rot is associated with cytosolic calcium deficiency, which results from vacuolar calcium overload driven by the overexpression of transporter genes such as *MdCAX1* (Park et al., 2005; Tonetto De Freitas et al., 2011; Han et al., 2022). In contrast, under unfavorable conditions, mango (*Mangifera indica*) spongy tissue leads to abnormal calcium accumulation in cell walls and vacuoles, thereby depleting functional calcium in the cytosol (Ma et al., 2023). Collectively, these phenomena indicate that disturbances in calcium homeostasis are a well‐documented cause of various physiological disorders in fruits. In fruits, CAX dysfunction leads to localized calcium deficiency and physiological disorders. In Arabidopsis, it directly regulates calcium signaling dynamics, affecting both its initiation and termination. The deeper link between these symptoms and the signaling pathways warrants further investigation.

## CONCLUSION AND PERSPECTIVE

**Figure 5:**
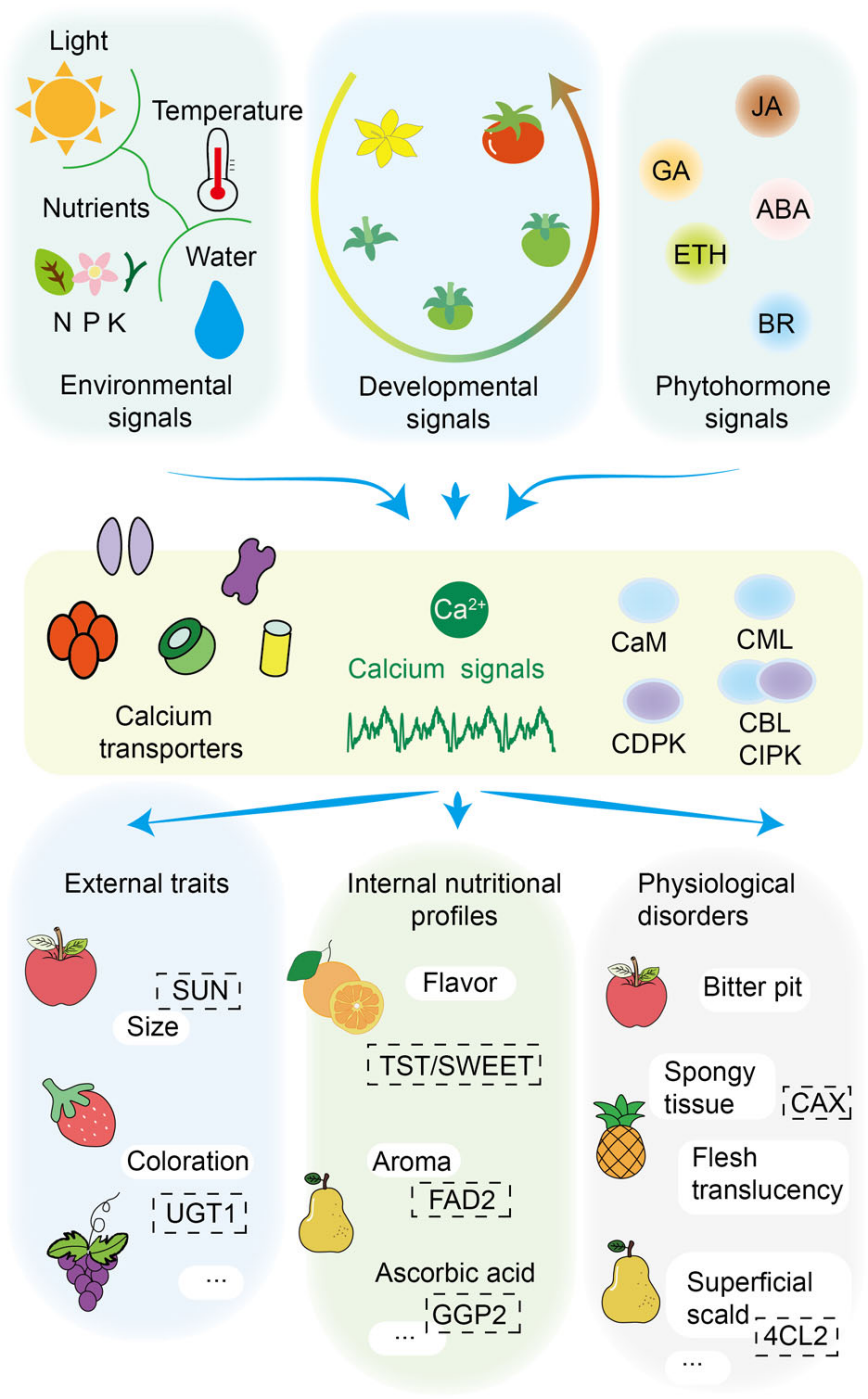
Calcium signaling acts as a central hub, integrating internal and external signals to orchestrate the multidimensional regulation of fruit appearance, nutritional quality, and physiological homeostasis. Fruit drawings were drawn by the author using Adobe Illustrator, based on reference images available online.

Calcium is an essential nutrient for plants and plays a crucial role in their growth and development. This review provides a systematic exposition of the role of calcium in determining fruit quality (Figure 5). However, the efficacy of these functions depends on efficient calcium uptake from the soil and its subsequent translocation to developing fruits, processes that often face substantial bottlenecks (Sumedrea et al., 2021; Cardona et al., 2023; Hirzel, 2023). Soil application is hampered by low absorption efficiency due to soil pH (Liang et al., 2024), nutrient antagonism (Li et al., 2013), and the poor mobility of calcium within the plant, which can lead to physiological disorders in fruits. To overcome these limitations, several strategies have been developed. These include the direct supplementation of calcium via foliar spraying (Martins et al., 2020; Michailidis et al., 2021) or fruit‐directed applications (Zhang et al., 2019c). While fruit‐directed application is theoretically more efficient in directly increasing fruit calcium content, foliar spraying is more widely adopted in practice due to its greater operational convenience, lower cost, reduced risk of fruit skin damage, and broader applicability across various cropping systems. Other important strategies comprise the use of advanced formulations like nano‐calcium fertilizers for improved efficiency (Ranjbar et al., 2019; Zhu et al., 2023), the indirect enhancement of calcium content through plant growth regulators (Tonetto De Freitas et al., 2014), and the innovative genome editing of calcium transporter genes to fundamentally improve calcium accumulation (Park et al., 2005).

These diverse supplementation strategies are ultimately designed to harness two fundamental roles of calcium: as a structural component, stabilizing cell walls and membranes to enhance mechanical strength and extend shelf life (Huang et al., 2023), and as a versatile signaling molecule, regulating the biosynthesis of important compounds such as anthocyanins and AsA (Figure 5). However, current research faces three main limitations. First, most studies remain at the level of observing phenotypic changes after calcium treatment, lacking in‐depth analysis of the underlying mechanisms. Second, the intrinsic link between calcium treatment and the generation of endogenous calcium signals remains elusive. For instance, although a previous study reported rapid calcium oscillations in Arabidopsis guard cells induced by exogenous calcium (Weinl et al., 2008), and subsequent research has detected cytosolic calcium signals in protoplasts isolated from apple flesh tissue (Qiu et al., 2020), the mechanism by which cells accurately decode such externally induced signals and translate them into specific physiological responses remains to be clarified (Weinl et al., 2008). Finally, research focus has been excessively placed on downstream calcium sensors, while studies on calcium channels, the direct sources of calcium signals, are relatively scarce, particularly those on organelle membranes. Although studies have shown that AtCAX2 on the tonoplast can modulate the amplitude and duration of cytosolic calcium signals and thus contribute to waterlogging responses (Pittman et al., 2005), this remains indirect evidence. Whether and how calcium release channels such as TPC1 participate in signal transduction remains controversial in horticultural fruits (Islam et al., 2010). To move beyond the acknowledgment that decoding mechanisms are unclear, a concrete experimental strategy is proposed. First, employ Ca^2+^‐reporter transgenics to define the precise spatiotemporal ‘calcium signature’ associated with the stimulus. Second, perform genetic validation to establish necessity, starting with calcium channel mutants and extending to the putative decoder protein. Finally, use phospho‐proteomics to compare these key mutants with wild‐type plants under stimulus conditions and identify the specific substrate phosphorylation events that are abolished, thereby mapping the decoding pathway from signal influx to downstream response.

While the precise mechanisms of calcium signal generation and decoding remain to be clarified, significant progress has been made in understanding how the calcium signaling network interacts with phytohormone pathways to orchestrate fruit quality formation (Figure 5). Calcium interacts with ETH, JA, ABA, and IAA in a complex but coordinated manner, as exemplified by its role in ripening‐delaying with ETH in apple (Xu et al., 2023; Bianchetti et al., 2024), coloration‐interacting with MeJA variably in different fruits (Martins et al., 2018; Yu et al., 2020b), and development‐synergizing with auxin (Zhang et al., 2022). Understanding this calcium‐hormone network is key to developing precise strategies for quality control with reduced reliance on exogenous hormones (Yu et al., 2025). Beyond integrating internal hormonal cues, calcium signaling also functions as a master regulator decoding external environmental stimuli (Liang et al., 2022), such as abiotic stress, which profoundly impacts fruit quality. Research demonstrates that under various stress conditions, just as cold stress promotes the synthesis of anthocyanins, flavonoids, and AsA (Lin et al., 2025), drought stress increases SSC, which can be mediated by ABA (Zhu et al., 2024b), and high light intensity enhances carotenoid levels (Sunitha et al., 2019). Production practices have shown that calcium fertilizer application under moderate stress conditions can effectively enhance fruit quality formation (Feng et al., 2023; Naz et al., 2024). These findings provide a crucial theoretical foundation for the “stress cultivation” paradigm by utilizing calcium. Considering the intricate interplay between calcium and other signals, such as hormones, environmental cues, and developmental factors, future research could employ multi‐omics strategies, including metabolomics (Karakas et al., 2025), proteomics, and spatial transcriptomics, to systematically elucidate the complex mechanisms through which calcium collaboratively regulates fruit quality with multiple signaling factors. This approach will also provide crucial support for improving fruit quality.

## GENERATIVE AI USAGE STATEMENT

During the preparation of this work, the authors utilized DeepSeek (a generative AI tool by DeepSeek Company) for the purpose of polishing and improving the language and readability of the manuscript. After using this tool, the authors reviewed and edited the content as needed and took full responsibility for the final content of the publication.

## CONFLICTS OF INTEREST

The authors declare no conflicts of interest.

## AUTHOR CONTRIBUTIONS

C.L. and F.J. conceived and designed the review outline. F.J. wrote the manuscript draft with the help of S.G., M.L., Z.Z., and C.Y. C.L. and J.H.L. finalized writing and revision of the review manuscript. All authors have read and approved the contents of this paper.
